# The disruptive potential of data publication

**DOI:** 10.1098/rsnr.2016.0036

**Published:** 2016-08-24

**Authors:** Sabina Leonelli

**Affiliations:** University of Exeter, Byrne House, St German's Road, Exeter, Devon EX4 4PJ, UK

Scientific journals have long acted as a stabilizing force in academia, by defining scientific communities, demarcating subfields and showcasing their key insights. Stability derives not least from the structure of a scientific paper, which imposes order on the ever-shifting processes of data collection and analysis by fitting results into a systematic and cohesive narrative, aimed at persuading readers of the validity of a given knowledge claim. This is what philosophers of science have dubbed ‘reconstruction’ or ‘justification’ of scientific insights: papers provide an account that excludes research aspects and outputs that are not directly relevant to the arguments at hand—such as experiments or models that failed, data that proved irrelevant or neutral with regards to the hypotheses at stake, and procedural details that do not fit existing formats.^1^ Journal publications thus provide science with a quantifiable, fixed and spatio-temporally located output, which helps to adjudicate the success, and disseminate the achievements, of a given research effort.

The characteristics that make journal publications such crucial components of science can also, however, constitute a barrier to the dissemination of, and debate around, more fluid and dynamic aspects of research practice. I argue here that the history of data science, contemporary developments in data dissemination, and particularly current debates around making data freely and widely accessible and re-usable (‘Open Data’) and being able to integrate and analyse large datasets from a variety of sources (‘Big Data’) evidence an increasing dissatisfaction with the ‘orderly writing’ promoted by long-running journals such as *Philosophical Transactions*, and a growing fascination with documenting the evidence base of claims made in scientific papers in a way that facilitates questioning and re-interpretation.^2^ This is a major factor underlying the blossoming of publication models that differ considerably from the traditional journal, including entities such as data journals, repositories for research software, code and models, and discussion forums devoted to methodological discussions on reproducibility and research protocols.

The increasing success of these initiatives, and the accompanying rebellion against the use of metrics relating to journal publication (such as impact factors) to measure research excellence and productivity herald, it might be argued, an altogether new approach to scientific publishing—one in which the goal is not to stabilize and order research findings, but rather to highlight and debate the processual, complex and dynamic aspects of scientific inquiry, including the various types of more or less obviously useful data produced by any one research effort. Furthermore, many such projects are involving amateur scientists not only in data collection, but also in the interpretation and maintenance of data. By opening up the research process to outsiders, these initiatives are disrupting the social and scientific order imposed by narratives used in journal publications, and encouraging a wide recognition of the provisional nature of scientific knowledge and the enormous efforts and diverse expertise that go into validating a claim. Data publications also challenge researchers to share and discuss their immediate outputs before they have been analysed, thus highlighting the labour involved in producing data and the variety of ways in which those data could serve as evidence for scientific claims.

The Data Studies group at the University of Exeter (www.datastudies.eu), which includes philosophers, historians and sociologists of science, investigates the management and dissemination of research data across time, disciplines and geographical locations. Our research documents a disjuncture between the ways in which research data are moved and interpreted across research sites, and the monistic interpretation that they are given in scientific papers.^3^ Much of the Open Science discussion revolves around finding ways to disseminate data independently of papers, thus arguably enhancing their evidential value towards the production of new claims. Online digital infrastructures, such as databases, are typically regarded as key tools to address these concerns. At the same time, our research shows that the classification and visualization activities involved in developing a data infrastructure are highly sophisticated and require various types of expertise (including discipline-specific knowledge and information science).^4^ It is not yet clear how best to harness such expertise, and make it visible and publicly accessible.

Data journals are a possible solution: they make datasets citable and provide an important forum for critical discussion of data handling practices, including the activities of database curators. However, rethinking scientific publication as focusing on data has its own shortcomings. As the Royal Society has stressed, data access needs to be ‘intelligent’ and involve information about the conditions under which data have been gathered and circulated.^5^ Furthermore, there are no obvious criteria for refereeing data articles, and there is a danger of making scientific publications even more inaccessible to the general public (*pace* Open Data rhetoric, data publication tends to lack contextualization: having access to lots of scientific data is different from having the ability to use them). It is also not clear that ‘authorship’ is a useful way to think about ownership claims for data, particularly given the transformations that data may undergo as they are formatted, selected, manipulated and visualized across data infrastructures—and the inclusion of data gathered by citizen science initiatives.^6^

What is the role of scientific journals in such a landscape, and in which sense can or should they continue to bring stability and order to knowledge production? This is a question that learned societies, journal editors and publishers need to consider carefully, so as to take advantage of the opportunities involved in challenging and re-inventing scientific writing and the dissemination of research results.

## Notes

1 H. Reichenbach, *Experience and prediction: an analysis of the foundations and the structure of knowledge* (University of Chicago Press, 1938); T. Nickels, ‘Reconstructing science: discovery and experiment’, in *Theory and experiment* (ed. D. Batens and J. P. van Bendegem), pp. 33–53 (Reidel, Dordrecht and Boston, 1988).

2 J. R. Topham, ‘The scientific, the literary, and the popular: commerce and the reinvention of the scientific journal in Britain, 1815–25’, presentation at the Royal Society Symposium, The end of the scientific journal? Transformations in publishing (27 November 2015).

3 S. Leonelli, *Data-centric biology: a philosophical study* (University of Chicago Press, 2016).

4 S. Leonelli, ‘Documenting the emergence of bio-ontologies: or, why researching bioinformatics requires HPSSB’, *Hist. Phil. Life Sci.* **32**, 105–126 (2010); J. Kallinikos and N. Tempini, ‘Patient data as medical facts: social media practices as a foundation for medical knowledge creation’, *Inform. Syst. Res.* **25**, 817–833 (2014).

5 Royal Society, *Science as an open enterprise* (Royal Society, London, 2012).

6 R. A. Ankeny and S. Leonelli, ‘Valuing data in postgenomic biology: how data donation and curation practices challenge the scientific publication system’, in *Postgenomics*: *perspectives on biology after the genome* (ed. S. Richardson and H. Stevens), pp. 126–149 (Duke University Press, Durham, NC, 2015).

